# Evaluation of the Spanish population coverage of a prospective HLA haplobank of induced pluripotent stem cells

**DOI:** 10.1186/s13287-021-02301-0

**Published:** 2021-04-13

**Authors:** Belén Álvarez-Palomo, Iris García-Martinez, Jorge Gayoso, Angel Raya, Anna Veiga, María Luisa Abad, Adolfo Eiras, María Guzmán-Fulgencio, Mar Luis-Hidalgo, Cristina Eguizabal, Silvia Santos, Antonio Balas, Raquel Alenda, Francisco Sanchez-Gordo, Laura Ponce Verdugo, Juliana Villa, Enric Carreras, Francisco Vidal, Alejandro Madrigal, María José Herrero, Francesc Rudilla, Sergi Querol

**Affiliations:** 1 Banc de Sang i Teixits, Edifici Dr. Frederic Duran i Jordà, Passeig Taulat 116, 08005 Barcelona, Spain; 2 Musculoskeletal Tissue Engineering Group, Vall d’Hebron Research Institute (VHIR), Passeig de la Vall d’Hebron 129-139, 08035 Barcelona, Spain; 3 Organización Nacional de Trasplantes, Ministerio de Sanidad, C/ Sinesio Delgado 6 - Pabellón 3, 28029 Madrid, Spain; 4 Programa de Medicina Regenerativa, Institut d’Investigació Biomèdica de Bellvitge. IDIBELL, Hospital Duran i Reynals, Gran Via de l’Hospitalet, 199-203, 08908 L’Hospitalet de Llobregat, Barcelona, Spain; 5 Axencia Galega de Sangue, Órganos e Tecidos, Rúa Xoaquín Díaz de Rábago 2, 15705 Santiago, Spain; 6 Centro de Transfusión de la Comunidad Valenciana, Av. del Cid, 65-acc, 46014 Valencia, Spain; 7 Basque Center for Blood Transfusion and Human Tissues, Barrio Labeaga, s/n, 48960 Galdakao, Spain; 8 Cell Therapy, Stem Cells and Tissues Group, Biocruces Bizkaia Health Research Institute, Células Madre y Tejidos, Cruces Plaza, 48903 Barakaldo, Spain; 9 Centro de Transfusión de la Comunidad de Madrid, Avda. de la Democracia, s/n, 28032 Madrid, Spain; 10 Centro de Transfusión, Tejidos y Células de Málaga, Avda. Doctor Gálvez Ginachero s/n, 29009 Málaga, Spain; 11 REDMO / Fundació i Institut de Recerca Josep Carreras, C/Muntaner, 383 2n, 08021 Barcelona, Spain; 12 Transfusional Medicine, Vall d’Hebron Research Institute, Universitat Autònoma de Barcelona (VHIR-UAB), Barcelona, Spain; 13 CIBER de Enfermedades Cardiovasculares (CIBERCV), Madrid, Spain; 14 Royal Free Hospital, Pond Street, Hampstead, NW3 2QG UK; 15 UCL Cancer Institute, Medical School, 74 Huntley St, Bloomsbury, London, WC1E 6DE UK

**Keywords:** Induced pluripotent stem cells, Haplobank, HLA matching, Homozygous

## Abstract

**Background:**

iPSC (induced pluripotent stem cells) banks of iPSC lines with homozygous HLA (human leukocyte antigen) haplotypes (haplobanks) are proposed as an affordable and off-the-shelf approach to allogeneic transplantation of iPSC derived cell therapies. Cord blood banks offer an extensive source of HLA-typed cells suitable for reprogramming to iPSC. Several initiatives worldwide have been undertaken to create national and international iPSC haplobanks that match a significant part of a population.

**Methods:**

To create an iPSC haplobank that serves the Spanish population (IPS-PANIA), we have searched the Spanish Bone Marrow Donor Registry (REDMO) to identify the most frequently estimated haplotypes. From the top ten donors identified, we estimated the population coverage using the criteria of zero mismatches in HLA-A, HLA-B, and HLA-DRB1 with different stringencies: high resolution, low resolution, and beneficial mismatch.

**Results:**

We have calculated that ten cord blood units from homozygous donors stored at the Spanish cord blood banks can provide HLA-A, HLA-B, and HLA-DRB1 matching for 28.23% of the population.

**Conclusion:**

We confirm the feasibility of using banked cord blood units to create an iPSC haplobank that will cover a significant percentage of the Spanish and international population for future advanced therapy replacement strategies.

**Supplementary Information:**

The online version contains supplementary material available at 10.1186/s13287-021-02301-0.

## Introduction

Induced pluripotent stem cells (iPSC) hold great promise in the field of regenerative medicine due to their capacity to both self-renew and differentiate into any cell-type of the human body. Unlike embryonic stem cells, iPSC are free of ethical concerns and allow the autologous application of cell replacement therapies [1, 2]. There has been extensive research and development in the field to create safe iPSC and protocols to differentiate them into clinically relevant cells for cell therapy applications. This research effort culminated in 2015 with the first successful clinical trial in Japan that used iPSC derived retinal pigmented epithelial cells (RPE) in an age-related macular degeneration (AMD) patient (RIKEN trial) [3]. However, the high cost and lengthy process of iPSC production could make the use of iPSC prohibitive for many applications. Consequently, the idea of using clinically matched iPSC for HLA-A, HLA-B, and HLA- DRB1 as an allogeneic treatment became more widespread [4].

Although MHC (major histocompatibility complex) class I and II molecules are potentially immunogenic, HLA-A, HLA-B, and HLA–DRB1 are the strongest determinants of rejection of an allogeneic transplant [5]. The effect of HLA-A, HLA-B, and HLA-DRB1 mismatch on solid organ transplant rejection has been extensively documented [6]. It is important to note that HLA matching for solid organ transplantation mainly takes into account HLA-A, HLA-B, and HLA-DRB1 loci at a low-resolution level [7].

The level of HLA-matching requirements ranges depending on the type of transplant, with a minimum score of 9/10 (HLA-A, HLA-B, HLA-C, HLA-DRB1, HLA-DQB1) for bone marrow transplantation of unrelated donors, a minimum score of 6/8 (HLA-A, HLA-B, HLA-C, HLA-DRB1) for cord blood [8] to kidney transplants in which several degrees of “beneficial match” are considered, from one mismatch in HLA-A or HLA-B to just matching HLA-DRB1 alone [9]. Currently, for hematopoietic progenitor transplantation, the common use is high-resolution sequencing (two fields) of five HLA loci [10] using Next Generation Sequencing (NGS) for typing.

The relevance and stringency of HLA-matching for iPSC-derived cells for clinical transplantation has been discussed extensively [11]. Unlike transplantation of hematopoietic progenitors, iPSC derivatives do not have contaminating immune cells and therefore the level of compatibility required is measured only for host vs. graft because the graft does not have in this case the ability to react against the host mismatched HLA antigens. HLA-A, HLA-B, and HLA-DRB1 matching have shown to confer a clear advantage over totally allogeneic transplant, with different degrees of immunological responses observed indicating the need for immunosuppression [4, 12].

Banking of HLA-typed pluripotent cells for matching a wide proportion of a population was first proposed for embryonic stem cells [13]. The authors also proposed the use of homozygous donors for common HLA-A, HLA-B, and HLA-DRB1 haplotypes as a way to provide HLA match for a reasonable percentage of the target population with a limited number of cell lines. Later, with the appearance of iPSC, Nakatsuji and colleagues proposed the use of banked cord blood as a source of HLA-typed cells for the construction of HLA homozygous iPSC banks (haplobanks) [14]. It was estimated that a haplobank with only 30 iPSC lines would be able to cover 82.2% of the Japanese population and 50 lines, 90.7% [14]. Other studies have calculated the coverage of haplobanks for the UK population [15], South Korea [16], China [17], and the USA [18]. iPSC haplobanks created from cord blood and peripheral blood donors are already a reality in South Korea and Japan [16, 19, 20].

The HLA haplotype landscape in Spain has been investigated before with small cohorts of patients and healthy individuals [21–23] and a larger cohort of 5458 units of cord blood from the Barcelona Cord Blood Bank, HLA-typed in high resolution [24].

The Spanish project IPS-PANIA aims at creating an iPSC haplobank of at least seven clinical-grade lines to provide maximum coverage to the Spanish population. To identify the haplotypes providing maximum coverage (probability of zero mismatches in HLA-A, HLA-B, and HLA-DRB1), we have searched a large cohort of 32,000 adult bone marrow donors and calculated the estimated coverage for a study population of 418,981 individuals including cord blood donors plus bone marrow donors from REDMO registry. We have concluded that a haplobank of seven lines would cover 23.69% of the Spanish population and ten lines would cover 28.23%.

## Materials and methods

### Study cohort and ethics approval

The REDMO includes the HLA typing of all cord blood donations and all the adult bone marrow donors of the Spanish population. For population coverage studies, the target population consisted of all the cord blood plus all the adult bone marrow donors in REDMO. The consultation of the HLA data was approved by the Ethics Committee for Research with Medicines from Vall d’Hebron Hospital (Barcelona, Spain) and the Transplantation and Regenerative Medicine Commission of the Spanish National Health System.

### HLA typing and haplotype frequency determination

High-resolution typing was performed by Sanger sequencing in an ABI PRISM 3130xl Genetic Analyzer (Thermo Fisher) and/or NGS in a MiSeq platform (Illumina) or in Ion GeneStudio S5 System (Thermo Fisher) for *HLA-A*, *HLA-B*, *HLA-C*, *HLA-DRB1*, *HLA-DRB3/4/5*, *HLA-DQB1*, and *HLA-DPB1* genes. The resulting sequences were analyzed using Assign 4.7.1 (CareDX), Type Stream Visual (One Lambda), and NGSengine (GenDX, 2.16), depending on the used procedure. Low resolution was performed using a PCR-SSO (Luminex) based method for HLA-A, B, C, DRB1, and DQB1 genes.

The expectation-maximization algorithm implemented in the Arlequin software (version 3.5.2.2) [25] was used to estimate maximum-likelihood haplotype frequencies, considering the high-resolution (4-digit) allelic frequencies of three (A, B, and DRB1) and five HLA genes (A, B, C, DRB1, and DQB1), from 30,000 and 27,000, respectively, randomly selected adult subjects of the REDMO project.

### Screening and selection of HLA haplotype homozygous donors

All the cord blood units in REDMO were studied for the identification of potential HLA-A, HLA-B, and HLA-DRB1 homozygous cord blood donors. Selection and classification of homozygous units were performed by simple counts with Microsoft Excel.

### Calculation of match coverage

To estimate the Spanish population HLA matching coverage of a 10 iPSC haplobank, we counted the number individuals with zero mismatches in HLA-A, HLA-B, and HLA-DR when compared with the top ten haplotypes selected by frequency. The population cohort was composed of the combined data of the adult bone marrow donors and cord blood donors in the REDMO collection. The calculation was done either in two digits HLA typing (low plus high resolution originally) on 418.980 individuals or in four digits (only high resolution) on 56,798 individuals. The haplotype match benefit (coverage) in the whole sample (including adult bone marrow donors and cord blood donors) was estimated with an in-house, iterative algorithm in R. Briefly, in each iteration, the estimated haplotype that matched the highest number of subjects was identified. Then, the best haplotype and all matched individuals were extracted from the dataset and coverage was recomputed in the remaining data. The matching was based on the concordance of the A, B, and DRB1 alleles of the estimated homozygous haplotypes with at least one of the two alleles of these loci in each individual. To calculate the coverage allowing for mismatch in one or two alleles (beneficial match), the same strategy was applied, but one or two mismatches in any HLA loci was tolerated. In all cases, the cumulative percentage of coverage was calculated by dividing the number of matched individuals in each iteration by the total sample size, multiplied by 100.

## Results

### Haplotype frequencies

**Fig. 1:**
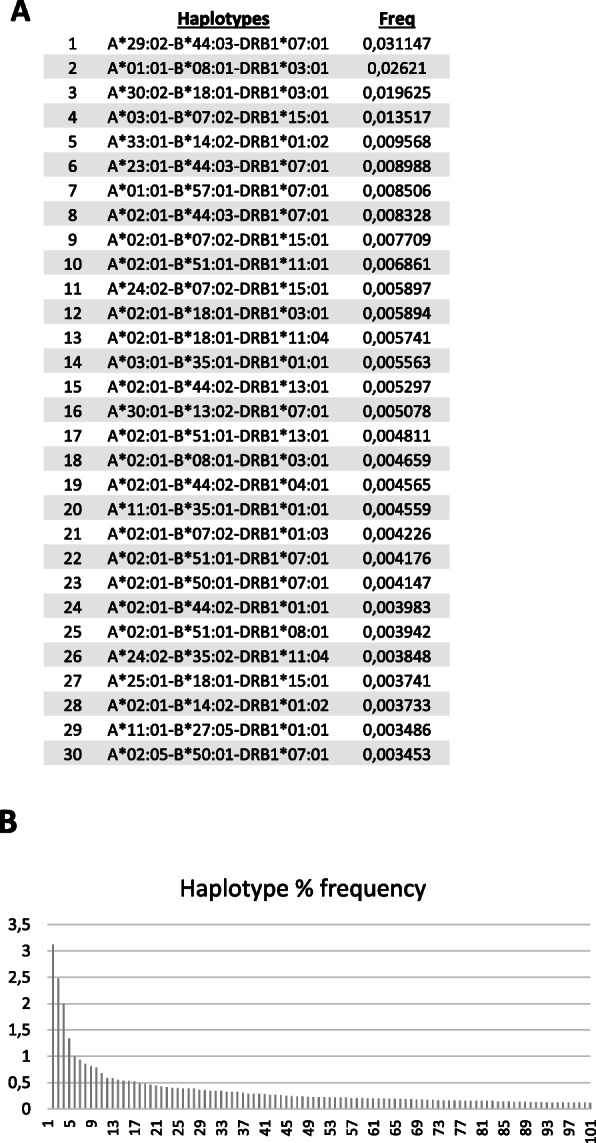
**a** Top 30 ranking HLA-A, HLA-B, and HLA-DRB1 estimated haplotypes by % of frequency at the Spanish Bone Marrow Donor Registry. **b** Graphic representation of the % of frequency of the top 100 HLA-A, HLA-B, and HLA-DRB1 estimated haplotypes

A cohort of 30,000 randomized high-resolution HLA typing adult bone marrow donors from the REDMO identified a total of 8478 different haplotypes for HLA-A, HLA-B, and HLA-DRB1 (Fig. 1a and Supplementary Excel file for the complete list). Five of them presented a frequency 1% or higher, namely A*29:02~B*44:03~DRB1*07:01 (3.12%), A*01:01~B*08:01~DRB1*03:01 (2.48%), A*30:02~B*18:01~DRB1*03:01 (1.99%), A*03:01~B*07:02~DRB1*15:01 (1.34%), and A*33:01~B*14:02~DRB1*01:02 (1.00%). Haplotype frequencies show a steep decline after the first five (Fig. 1b).

We performed the same analysis for five genes, namely HLA-A, HLA-B, HLA-C, HLA-DRB1, and HLA-DQB1 with a 27,000 cohort from the REDMO registry. Only the top six positions corresponded with those haplotypes identified in the three genes analysis and only four had a frequency above 1%: A*29:02~ C*16:01~B*44:03~DRB1*07:01~DQB1*02:02 (2.95%), A*01:01~C*07:01~B*08:01~DRB1*03:01~DQB1*02:01 (2.52%), A*30:02~C*05:01~B*18:01~DRB1*03:01~DQB1*02:01 (1.89%), and A*03:01~ C*07:02~B*07:02~DRB1*15:01~DQB1*06:02 (1.34%) (Sup. Figure 1).

### Identification of homozygous donors

**Fig. 2:**
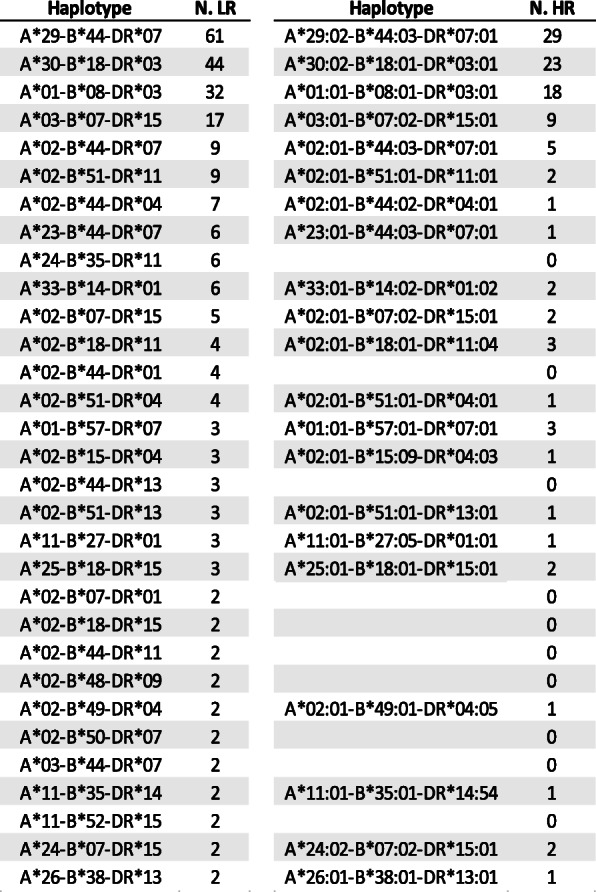
HLA haplotypes and number of units (N.) of HLA-A, HLA-B, and HLA-DRB1 homozygous found in the Spanish registry of public cord blood banks and present at least in two units. LR, low resolution; HR, high resolution

To identify homozygous cord blood units available in the Spanish banks that can be candidates as source cells to create the iPSC haplobank, we looked into the Spanish registry, which comprises 52,220 cord blood donations in Spain. HLA was typed in low resolution (serological and PCR based) for 42.801 units and high resolution (NGS) for 9419 units. We identified 322 cord blood units homozygous for HLA-A, HLA-B, and HLA-DRB1 (0.62% of the units) representing 111 different haplotypes (Sup. Figure 2). So far, 109 of the homozygous units were verified by high-resolution HLA typing and represented 43 different haplotypes. Not to deplete the cord blood banks of any haplotype for hematopoietic stem cell transplantation, we only considered those haplotypes with two or more units banked as candidates for the iPSC bank (Fig. 2). Thirty-one haplotypes were represented at least twice in low resolution and 11 in high resolution. The top ten positions in the most frequent haplotypes from the adult bone marrow donor study were all represented in at least two units genotyped in low resolution and nine of them have confirmed units by high resolution*.*

### Recipients matching coverage

**Fig. 3:**
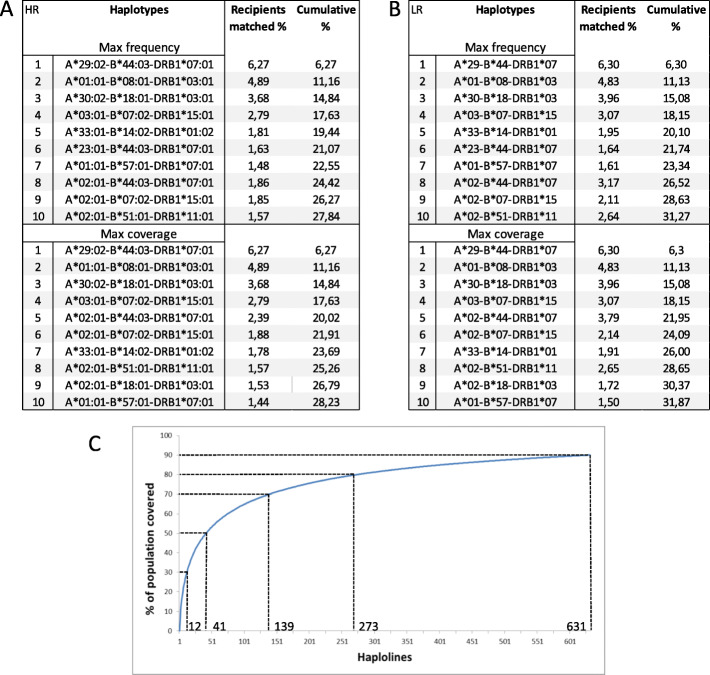
Estimated percentage and cumulative percentage of HLA-matched individuals in the Spanish population with a panel of 10 homozygous donors in high resolution (**a**) and low resolution (**b**). Estimated numbers of iPSC lines homozygous for HLA-A, HLA-B, and HLA-DRB1 (haplolines) to cover the Spanish population (**c**)

We tested the first 10 most frequent haplotypes for three genes, HLA-A, HLA-B, and HLA-DRB1, and we found that the cumulative coverage was 27.84% of the population in high resolution and 31.27% in low resolution (Fig. 3a, b). We also explored the “maximum coverage” approach for 10 haplolines selecting not the most frequent haplotypes but those that would optimize the coverage. The choice of haplotypes with this approach did not change for the top four positions, and the following six, although in a different order, only one new haplotype was considered: A*02:01~B*18:01~DRB1*03:01. The cumulative coverage in high resolution was slightly increased to 28.23% in high resolution and to 31.87% in low resolution (Fig. 3a, b). Since we did not identify any homozygous cord blood unit in the Spanish banks with A*02:01~B*18:01~DRB1*03:01, we estimated the optimized coverage considering only the available units and then the accumulated coverage in high resolution is 27.95% and 32.58% in low resolution. To calculate how many haplolines would be needed to cover close to 100% of the Spanish population, we repeated this iterative process with all the estimated haplotypes in our study population. We found that 100 haplolines would cover 65.94% of the Spanish population and that 630 haplolines would be necessary to cover 90% of the potential recipients (Fig. 3c). We also did this calculation with 5 genes: HLA-A, HLA-B, HLA-C, HLA-DRB1, and HLA-DQB1, and then the numbers shift to 100 units to cover 50% of the population and 897 units needed to cover 90% (Sup. Figure 3).

### Beneficial match

**Fig. 4:**
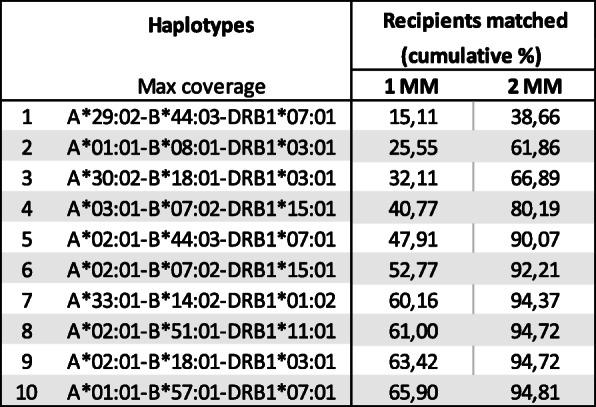
Estimated cumulative percentage of matched individuals in the Spanish population considering a “beneficial match” with a panel of 10 homozygous donors from Spanish cord blood Banks in high resolution allowing for one mismatch in any of the alleles HLA-A, HLA-B, or HLA-DRB1 (1 MM) or two mismatches (2 MM)

As different iPSC-derived cell types and different engraftment sites might present different HLA matching requirements, we introduced the approach of “beneficial match” and estimated the coverage in the case that one or two of the alleles studied did not match as the most immunogenic of the alleles (Fig. 4). The cumulative coverage for the top 10 HLA-A, HLA-B, and HLA-DRB1 haplotypes in the “maximum coverage” list showed a wide population coverage of 65.9% when allowing for one mismatch in any of the three alleles considered and a coverage of 94.81% when allowing to mismatch in two of the three alleles.

## Discussion

Currently, clinical-grade iPSC haplobanks are already available in South Korea and Japan [19, 20, 26] and similar initiatives are being undertaken in other countries such as Australia [27]. The effectiveness of the approach will come from a global collaborative to share haplolines and this implies an effort to standardize the production and quality control by the different banks [28, 29].

The IPS-PANIA project aims at developing a haplobank that can serve a significant percentage of the Spanish population with HLA-matching iPSC lines that can be used as starting material for iPSC-derived cell therapies. In the initial phase of the project, we have set a goal developing seven haplolines.

A previous study of the estimated most frequent haplotypes in the Spanish bone marrow donor database identified five haplotypes with a frequency ranging from higher than 1% to the 6th position in the list were the same as previously described for the Barcelona cord blood bank study [24]. The differences in the lower positions might reflect the higher accuracy of the larger database or geographical or generational differences. The haplotype with the top frequency is A*29:02~B*44:03~DRB1*07:01 and the third one A*30:02~B*18:01~DRB1*03:01 are very common in the western Mediterranean region. These haplotypes are less common in Italy and the rest of south-eastern Europe [30]. A*29:02~B*44:03~DRB1*07:01 is also a common haplotype in the Hispanic population in the USA, but less frequent for European ancestry and extremely rare for Asian ancestry [17, 31]. The second and fourth most frequent haplotypes, A*01:01~B*08:01~DRB1*03:01 and A*03:01~B*07:02~DRB1*15:01 respectively, are among the most common in the northern and central European population. From the top 10 most frequent haplotypes in Spain, seven are in the top 30 described for the UK [15]. As expected, they are not identified with the most frequent haplotypes identified for Korea and Japan [14, 16].

The identification of homozygous units confirmed that the Spanish public cord banks contained enough homozygous units that could be used to build an iPSC haplobank to cover the top haplotypes without compromising the availability of any lifesaving unit needed for hematopoietic progenitor transplantation. We found a 0.62% of the cord blood units were homozygous, a significantly lower number as compared for that reported for the South Korean population (0.79%), which might reflect the wider heterogeneity of the HLA genetics in the Spanish population.

The estimated population coverage of the most frequent haplotypes seems to also reflect a higher HLA genetic variability in the Spanish population as compared to other populations: the top haplotype in Spain covers 6.3% and together with the second most frequent haplotype, the cumulative coverage is 11.2%, while for Korea, the percentages are 9.2 and 14.5, and for UK, 16.9% and 26.4%. A ten cell line haplobank would cover 28% in four-field resolution and 32% in two-field resolution, while in UK or Japan, the reported coverages for ten cell lines are closer to 50% of the population. The “maximum coverage” approach for the selection of the top ten candidates, opposed to following just the higher haplotype frequency order, improved the coverage for the lower positions but the effect was almost lost when the ten haplolines were considered. When calculating how many haplotypes would be needed for almost complete coverage of the Spanish population, we found that 631 would be needed to cover 90%, again revealing a much wider variety of existing haplotypes as compared to other populations analyzed in other studies, such as Korea that found 90% covered with less than 200 cell lines [16].

Wider coverage of iPSC haplolines by lowering the HLA stringency might be worth consideration for an iPSC bank. It is still early days to know what the real HLA matching requirements of iPSC derived products will be as the first clinical trial carried out at the Kobe City Medical Center in collaboration with Osaka University, using allogeneic iPSC-derived retinal pigmented epithelial cells of a HLA-A, HLA-B, HLA-DRB1-matching haplotype has not been reported yet. It is clear that HLA matching is advantageous and will reduce the degree of immunosuppression although this will be determined by the type of cell and the transplantation sites. Studies performed in non-human primates matching MHC antigens equivalent to HLA-A, HLA-B, and HLA-DRB1 have revealed from mild immune infiltration in iPSC-RPE implanted in the retina [4] to a significant reaction to the allograft requiring immunosuppression in the central nervous system [10]. Unlike hematopoietic progenitors and organ transplants, iPSC-derived cells will be free of contaminating T cells, except for a small possibility in the case of hematological lineage derivation, and no graft-versus-host disease is expected, making sense to consider less HLA-match stringent scenarios closer to solid organ transplants like the kidney. The population coverage when considering haplotypes in two-field resolution was not much increased as compared to four-field resolution, highlighting the predominance of certain subgroups of haplotypes. When the beneficial match was considered, as expected, the gains were much greater when allowing for one or two mismatches in any of the three alleles. The choices of HLA matching stringency requirements will be determined by future evidence for the different cell types derived from iPSC and the different transplantation sites.

Taken together, a 30% population match for a ten cell line haplobank is a significant proportion of potential patients who may benefit from the cell bank. This justifies the construction of the Spanish haplobank, more so when considering a worldwide effort to share cells internationally with other banks that might contain less frequent haplotypes. Also, as the production of clinical-grade iPSC lines is optimized and streamlined, it will become easier and more affordable to increase the number of haplolines in the bank to reach several dozens and cover a much wider proportion of the population. With this study, we have investigated the feasibility to provide homozygous cord blood units to create an iPSC bank of a reduced number of haplolines that will serve a significant percentage of the Spanish and international population. Besides RPE cells for the treatment of AMD, several other iPSC-derived cells are presently being tested in clinical trials to treat conditions such as spinal cord injury, Parkinson’s disease, graft-versus-host-disease, heart failure, or cancer. Clinical-grade iPSC are intended to be used as starting material for future clinical trials and cell therapy products, accelerating the application of iPSC-based therapies soon.

## Conclusion

With the present study, we have been able to confirm that HLA homozygous cord blood units stored in Spanish cord blood banks can provide for the construction of an iPSC bank that is useful for a significant part of the Spanish population (about 28%). The haplotypes identified as providing the greatest coverage, may also be useful for other populations such as Europe and North America.

## Supplementary Information

**Additional file 1: Supplementary Figure 1.** Top 30 ranking HLA-A, -B, -C, -DRB1 and -DQB1 estimated haplotypes by % of frequency at the Spanish Bone Marrow Donor Registry.

**Additional file 2: Supplementary Figure 2.** HLA types and number of units (N.) of HLA-A, HLA-B and HLA-DRB1 homozygous found in the Spanish registry of public cord blood banks.

**Additional file 3: Supplementary Figure 3.** Estimated numbers of iPSC lines homozygous for HLA-A, -C, -B, -DRB1 and -DQB1 (haplolines) to cover the Spanish population.

**Additional file 4.**

## Data Availability

All the presented data is available for consultation.

## Abbreviations

AMD: Age-related macular degeneration
HLA: Human leukocyte antigen
iPSC: Induced pluripotent stem cells
NGS: Next Generation Sequencing
REDMO: Spanish Bone Marrow Donor Registry
RPE: Retinal pigmented epithelial cells
