# Supplementary material for: Evaluation of the Spanish population coverage of a prospective HLA haplobank of induced pluripotent stem cells
Source: Stem Cell Res Ther. 2021 Apr 13;12:233. doi: 10.1186/s13287-021-02301-0 (PMC8042859; doi:10.1186/s13287-021-02301-0)
Supplement: Supplementary file 1 — Additional file 1: Supplementary Figure 1. Top 30 ranking HLA-A, -B, -C, -DRB1 and -DQB1 estimated haplotypes by % of frequency at the Spanish Bone Marrow Donor Registry. [file 13287_2021_2301_MOESM1_ESM.pdf]

	Haplotypes	Freq
1	A*29:02-B*44:03-C*16:01-DRB1*07:01-DQB1*02:02	0,029456
2	A*01:01-B*08:01-C*07:01-DRB1*03:01-DQB1*02:01	0,025239
3	A*30:02-B*18:01-C*05:01-DRB1*03:01-DQB1*02:01	0,018859
4	A*03:01-B*07:02-C*07:02-DRB1*15:01-DQB1*06:02	0,014086
5	A*33:01-B*14:02-C*08:02-DRB1*01:02-DQB1*05:01	0,009598
6	A*23:01-B*44:03-C*04:01-DRB1*07:01-DQB1*02:02	0,008274
7	A*02:01-B*07:02-C*07:02-DRB1*15:01-DQB1*06:02	0,00789
8	A*24:02-B*07:02-C*07:02-DRB1*15:01-DQB1*06:02	0,005673
9	A*03:01-B*35:01-C*04:01-DRB1*01:01-DQB1*05:01	0,005565
10	A*02:01-B*18:01-C*05:01-DRB1*03:01-DQB1*02:01	0,005423
11	A*30:01-B*13:02-C*06:02-DRB1*07:01-DQB1*02:02	0,005085
12	A*02:01-B*44:03-C*16:01-DRB1*07:01-DQB1*02:02	0,00507
13	A*02:01-B*44:02-C*05:01-DRB1*13:01-DQB1*06:03	0,004941
14	A*11:01-B*35:01-C*04:01-DRB1*01:01-DQB1*05:01	0,004878
15	A*02:01-B*18:01-C*07:01-DRB1*11:04-DQB1*03:01	0,004646
16	A*02:01-B*08:01-C*07:01-DRB1*03:01-DQB1*02:01	0,004419
17	A*02:01-B*07:02-C*07:02-DRB1*01:03-DQB1*05:01	0,004248
18	A*02:01-B*44:02-C*05:01-DRB1*04:01-DQB1*03:01	0,004233
19	A*01:01-B*57:01-C*07:01-DRB1*07:01-DQB1*03:03	0,004231
20	A*24:02-B*35:02-C*04:01-DRB1*11:04-DQB1*03:01	0,00389
21	A*11:01-B*27:05-C*01:02-DRB1*01:01-DQB1*05:01	0,003848
22	A*02:01-B*44:02-C*05:01-DRB1*01:01-DQB1*05:01	0,003806
23	A*02:01-B*50:01-C*06:02-DRB1*07:01-DQB1*02:02	0,003757
24	A*25:01-B*18:01-C*12:03-DRB1*15:01-DQB1*06:02	0,003729
25	A*26:01-B*38:01-C*12:03-DRB1*13:01-DQB1*06:03	0,003539
26	A*02:01-B*14:02-C*08:02-DRB1*01:02-DQB1*05:01	0,003246
27	A*33:01-B*14:02-C*08:02-DRB1*03:01-DQB1*02:01	0,003203
28	A*01:01-B*57:01-C*06:02-DRB1*07:01-DQB1*03:03	0,003049
29	A*02:05-B*50:01-C*06:02-DRB1*07:01-DQB1*02:02	0,003005
30	A*02:01-B*49:01-C*07:01-DRB1*01:01-DQB1*05:01	0,002987
