# Supplementary material for: Evaluation of the Spanish population coverage of a prospective HLA haplobank of induced pluripotent stem cells
Source: Stem Cell Res Ther. 2021 Apr 13;12:233. doi: 10.1186/s13287-021-02301-0 (PMC8042859; doi:10.1186/s13287-021-02301-0)
Supplement: Supplementary file 2 — Additional file 2: Supplementary Figure 2. HLA types and number of units (N.) of HLA-A, HLA-B and HLA-DRB1 homozygous found in the Spanish registry of public cord blood banks. [file 13287_2021_2301_MOESM2_ESM.pdf]

HLA type	N.
A29-B44-DR07	61
A30-B18-DR03	44
A01-B08-DR03	32
A03-B07-DR15	17
A02-B44-DR07	9
A02-B51-DR11	9
A02-B44-DR04	7
A23-B44-DR07	6
A24-B35-DR11	6
A33-B14-DR01	6
A02-B07-DR15	5
A02-B18-DR11	4
A02-B44-DR01	4
A02-B51-DR04	4
A01-B57-DR07	3
A02-B15-DR04	3
A02-B44-DR13	3
A02-B51-DR13	3
A11-B27-DR01	3
A25-B18-DR15	3
A02-B07-DR01	2
A02-B18-DR15	2
A02-B44-DR11	2
A02-B48-DR09	2
A02-B49-DR04	2
A02-B50-DR07	2
A03-B44-DR07	2
A11-B35-DR14	2
A11-B52-DR15	2
A24-B07-DR15	2
A26-B38-DR13	2
A01-B07-DR04	1
A01-B08-DR04	1
A01-B37-DR07	1
A01-B40-DR14	1
A01-B44-DR07	1
A01-B51-DR13	1
A01-B57-DR04	1
A01-B57-DR13	1
A02-B07-DR07	1
A02-B08-DR03	1
A02-B14-DR01	1
A02-B14-DR03	1
A02-B14-DR07	1
A02-B15-DR13	1
A02-B27-DR04	1
A02-B35-DR09	1
A02-B35-DR14	1
A02-B38-DR13	1
A02-B39-DR04	1
A02-B39-DR13	1
A02-B39-DR14	1
A02-B40-DR13	1
A02-B44-DR08	1
A02-B48-DR08	1
A02-B49-DR11	1
A02-B49-DR13	1
A02-B51-DR07	1
A02-B51-DR08	1
A02-B52-DR15	1
A02-B53-DR10	1
A02-B58-DR13	1
A03-B07-DR13	1
A03-B27-DR16	1
A03-B35-DR01	1
A03-B38-DR13	1
A03-B39-DR13	1
A11-B07-DR15	1
A11-B18-DR11	1
A11-B35-DR01	1
A11-B40-DR04	1
A11-B44-DR07	1
A11-B49-DR13	1
A11-B51-DR04	1
A23-B44-DR03	1
A24-B08-DR03	1
A24-B14-DR01	1
A24-B15-DR14	1
A24-B18-DR15	1
A24-B35-DR01	1
A24-B35-DR04	1
A24-B35-DR16	1
A24-B38-DR13	1
A24-B38-DR15	1
A24-B40-DR04	1
A24-B41-DR03	1
A26-B40-DR13	1
A29-B44-DR01	1
A29-B44-DR11	1
A29-B44-DR15	1
A29-B45-DR15	1
A29-B57-DR01	1
A30-B07-DR15	1
A30-B13-DR07	1
A30-B15-DR15	1
A30-B27-DR15	1
A30-B53-DR15	1
A31-B35-DR04	1
A31-B38-DR14	1
A32-B14-DR07	1
A32-B35-DR07	1
A33-B14-DR03	1
A33-B14-DR07	1
A33-B18-DR03	1
A33-B44-DR01	1
A66-B41-DR13	1
A66-B52-DR15	1
A68-B14-DR13	1
A68-B44-DR11	1
