# Supplementary figures and images for: Evaluation of the Spanish population coverage of a prospective HLA haplobank of induced pluripotent stem cells
Source: Stem Cell Res Ther. 2021 Apr 13;12:233. doi: 10.1186/s13287-021-02301-0 (PMC8042859; doi:10.1186/s13287-021-02301-0)

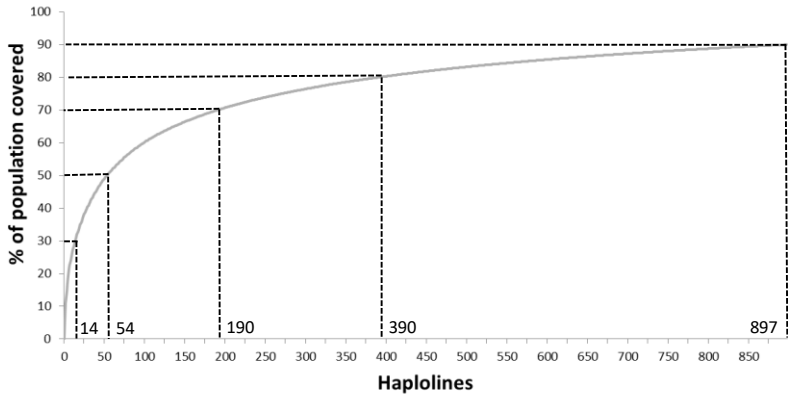

Supplement: Supplementary file 3 — Additional file 3: Supplementary Figure 3. Estimated numbers of iPSC lines homozygous for HLA-A, -C, -B, -DRB1 and -DQB1 (haplolines) to cover the Spanish population. [file 13287_2021_2301_MOESM3_ESM.pdf]
